# Anorectal Melanoma: A Case Report and an Update of a Rare Malignancy

**DOI:** 10.14740/wjon864w

**Published:** 2015-02-14

**Authors:** Waqas Jehangir, Nicole Schlacter, Shilpi Singh, Souad Enakuaa, Mohammed A. Islam, Shuvendu Sen, Abdalla Yousif

**Affiliations:** a Raritan Bay Medical Center, Perth Amboy, NJ 08861, USA; b Rutgers University School of Health Related Professions, Piscataway, NJ 08854, USA

**Keywords:** Melanoma, Anorectal, Malignancy, Wide local excision, Abdominal perineal resection

## Abstract

Anal melanoma is an aggressive but rare malignancy. Patients commonly present with very advanced or even metastatic disease. Risk factors for anal melanoma are family history and an activating mutation of C-KIT. Surgical excision remains the mainstay of therapy. The presence of activating mutations of C-KIT has prompted use of C-KIT inhibitors such as imatinib and sunitini. Early diagnosis and treatment remain crucial. Abdominal perineal resection (APR) offers a higher rate of local control whereas wide local excision (WLE) can yield superior long-term survival.

## Introduction

Anorectal melanoma is an extremely rare malignancy with a high metastatic potential and equally high mortality rate [1]. Of all melanomas, those affecting the anorectal canal comprise only 1%. This constitutes only 0.5-2% of all anorectal malignancies [2]. It occurs more often in the fifth to eighth decades and is seen more frequently in the female population [1, 2]. The overall incidence of anorectal melanomas is 0.4% per million with an almost two times increased incidence in the Caucasian population compared with the African American [2].

Most patients afflicted with this malignancy present late in the course of the disease with non-specific complaints. In a case series of 18 patients, the most common presenting symptom was bright red blood per rectum [1]. Other common presenting complaints include rectal pain, change in bowel habits, presence of a rectal mass, non-bloody rectal discharge, anemia, weight loss, tenesmus and incontinence [1, 3]. Due to the lack of specificity of the presenting complaints, many patients are misdiagnosed upon initial evaluation [1, 3]. Common misdiagnoses include hemorrhoids, polyps, adenocarcinoma or ulcers [1, 3].

## Case Report

A 79-year-old female with past medical history of diverticulosis, coronary artery disease, hypertension, varicose veins and diabetes, presented with bleeding per rectum for the last 4 months. The patient reported noting the blood while straining during a bowel movement. Few days prior to admission, the patient went to her primary care physician who diagnosed her with hemorrhoids and prescribed rectal suppositories. The patient reported that her problem persisted despite the suppositories. On the day of admission, she had sudden frank bleeding per rectum with severe abdominal and chest pain. She also felt a mass coming out along with the blood and tried unsuccessfully, to push the mass back in. She felt extreme pain which was associated with shortness of breath, lightheadedness and dizziness. She denied any fever, chills, blurry vision, cough, nausea, vomiting or diarrhea. She does not have any allergies or any family history of cancer. She denied any smoking, alcohol use, or drug use history.

On physical exam, the patient was not in any acute distress with a PR 96/min, BP 110/52 mm Hg, temperature 98.6 °F, and RR 20/min. There was no pallor or jaundice present. Abdomen was soft, non-tender, non-distended, and bowel sounds present with no hepatosplenomegaly. Rectal exam revealed a dark colored mass measuring about 4 × 5 × 3 cm in size with a visible overlying blood clot. The mass was tender to palpation but had no active bleeding. Laboratory analysis showed Hb 11.8 g/dL, Hct 36.2%, WBC 8,400/μL, platelets 323,000,000/μL and comprehensive metabolic panel was completely normal. Chest X-ray was within normal limits.

**Figure 1:**
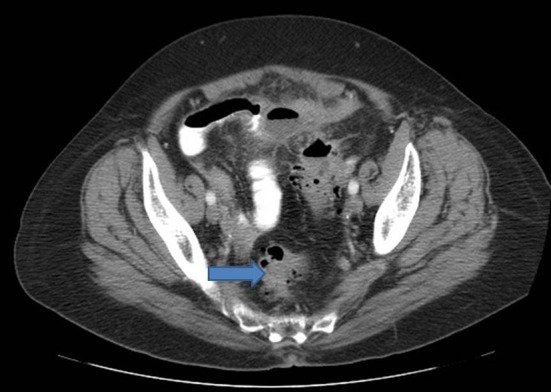
CT scan of abdomen showing thickening of the rectal mucosa.

**Figure 2:**
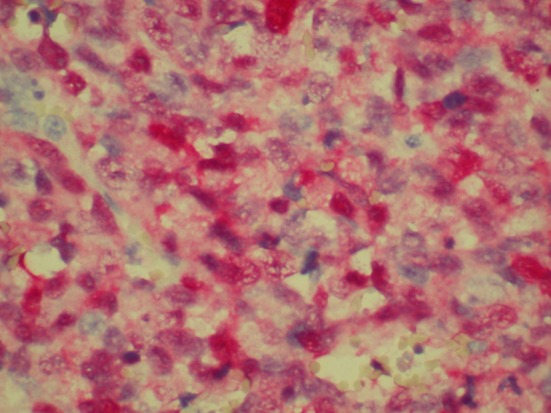
Biopsy of rectal mass positive for S-100.

**Figure 3:**
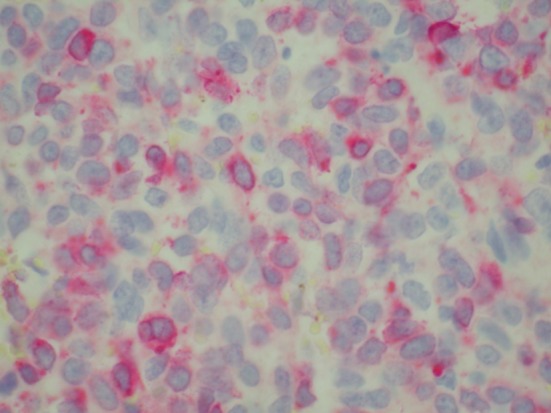
Biopsy of rectal mass positive for melan-A.

**Figure 4:**
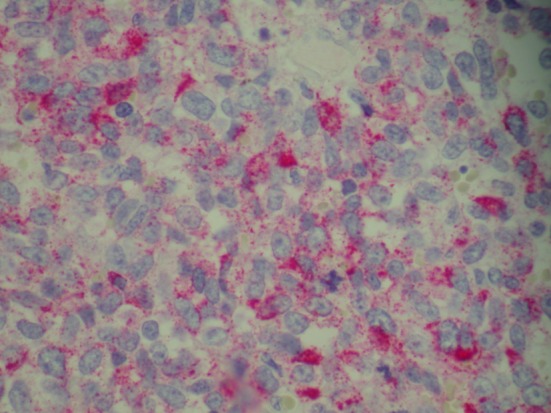
Biopsy of rectal mass positive for HMB-45.

Patient was admitted for prolapsed hemorrhoids. Surgical consult was called and a CT scan of abdomen/pelvis was performed which showed colonic diverticulosis with no acute diverticulitis. The rectal mucosal appeared thickened compared with the remainder of the colon and it was advised that the possibility of a rectal or other colonic neoplasm be excluded (Fig. 1). Biopsy of the rectal mass was performed which showed malignant melanoma. Immunohistochemical stain performed showed the tumor cells to be positive for S-100, melan A and HMB-45 (Fig. 2-4) and negative for CD34, chromogranin, synaptophysin, CD20, AE1/3, CK20, CD3 and CK7. The patient was diagnosed as primary mucosal malignant melanoma. Whole body scan did not reveal any metastasis. Patient was referred to cancer center for further treatment.

## Discussion

Anorectal melanoma is the third most common melanoma and it is the most common primary melanoma of the gastrointestinal tract [2]. Risk factors for this malignancy differ from those for cutaneous melanomas. Currently, identified risk factors include family history and an activating mutation of C-KIT [4]. It has been determined that the majority of anorectal melanomas originate from normal melanocytes in the intestinal epithelium [2, 5]. During fetal development, neural crest cells differentiate into melanocytes which then migrate throughout the body [5]. These melanocytes take up residence in the skin, eyes and mucosa [5]. In the rectum in particular, melanocytes are typically found at the anal transition zone and squamous zone [5].

Prompt diagnosis of anorectal melanomas is critical due to its highly aggressive nature. A mass may be identified on sigmoidoscopy or colonoscopy but is often initially misdiagnosed as other anorectal malignancies [5]. Computed tomography (CT) and MRI are the most accurate imaging modalities to determine the composition of the neoplasm and assess the extent of regional and distant spread [5]. Of the two, MRI is preferred for evaluating extraluminal spread of the malignancy [5]. On CT anorectal melanomas appear as bulky lesions that obscure the lumen on the rectum but unlike rectal carcinomas, do not obstruct it [5]. Endoscopic endorectal ultrasound is of important prognostic value because of its capability of determining tumor thickness and nodal involvement [5]. Once a biopsy is obtained, multiple immunohistochemical markers can be used to characterize anorectal melanomas including S-100 protein, melan A, HMB-45, tyrosinase and vimentin [2, 5].

Due to its rare nature, there is a lack of evidence from randomized control trials regarding appropriate treatment of anorectal melanomas [2]. Historically and to this day, however, the cornerstone of therapy remains surgical excision [2]. Two procedures are commonly performed in patients with resectable disease: wide local excision (WLE) or abdominoperineal resection (APR). The more conservative of the two approaches, WLE removes the neoplasm with clean margins of at least 10 mm, as indicated by histopathology [4]. In one study, WLE was associated with higher recurrence rates when compared with the more radical approach of APR [4]. On the other hand, studies have also shown that patients who underwent WLE had longer survival times. This result may be influenced by the smaller tumor burden and less advanced stage of disease found in patients undergoing WLE [4].

The role of medical therapy in the treatment of anorectal melanomas is another topic of debate. Little data exist to suggest its effectiveness; however, as more is uncovered about the pathophysiology of the malignancy, targeted therapies are being utilized. By far the most commonly used chemotherapy agent is dacarbazine [5]. Immunotherapy modalities such as IL-2 and ipilimumab have proven effective in some patients with unresectable disease [4]. The presence of activating mutations of C-KIT has prompted investigation into the use of C-KIT inhibitors such as imatinib, sunitinib, dasatinib, nilotinib and masatinib [4]. While these agents seem promising, it is important to consider the patient’s clinical status and degree of tumor burden due to their delay in onset [6].

Despite excision and adjuvant radiotherapy, anorectal melanomas carry a bleak prognosis. This is contributed in part to the non-specific complaints that patients present with and the high propensity of metastasis. In a patient case series of anorectal melanoma, the most common sites of metastasis were to the liver, lungs and pelvis [1]. Prognostic factors include lymph node involvement, extent of spread and tumor thickness [3]. The overall five-year survival of anorectal melanoma is less than 20% with a median survival of only 2 years [4].

A heightened awareness of the presentation of anorectal melanomas amongst practitioners will be critical to more prompt diagnoses and improved clinical outcomes. Further research is also indicated to better delineate appropriate therapeutic course of this silent malignancy.
